# CoS_2_/TiO_2_ Nanocomposites for Hydrogen Production under UV Irradiation

**DOI:** 10.3390/ma12233882

**Published:** 2019-11-24

**Authors:** Sivagowri Shanmugaratnam, Dhayalan Velauthapillai, Punniamoorthy Ravirajan, Alfred Antony Christy, Yohi Shivatharsiny

**Affiliations:** 1 Faculty of Engineering and Science, Western Norway University of Applied Sciences, 5020 Bergen, Norway; sivagowrishanmugaratnam@gmail.com; 2 Clean Energy Research Laboratory, Department of Physics, University of Jaffna, Jaffna 40000, Sri Lanka; pravirajan@gmail.com; 3 Department of Natural Science, University of Agder, 4630 Kristiansand, Norway; alfred.christy@uia.no; 4 Department of Chemistry, University of Jaffna, Jaffna 40000, Sri Lanka

**Keywords:** transition metal chalcogenides, titania, hydrothermal, hydrogen, water splitting

## Abstract

Transition metal chalcogenides have intensively focused on photocatalytic hydrogen production for a decade due to their stronger edge and the quantum confinement effect. This work mainly focuses on synthesis and hydrogen production efficiencies of cobalt disulfide (CoS_2_)-embedded TiO_2_ nanocomposites. Materials are synthesized by using a hydrothermal approach and the hydrogen production efficiencies of pristine CoS_2_, TiO_2_ nanoparticles and CoS_2_/TiO_2_ nanocomposites are compared under UV irradiation. A higher amount of hydrogen production (2.55 mmol g^−1^) is obtained with 10 wt.% CoS_2_/TiO_2_ nanocomposite than pristineTiO_2_ nanoparticles, whereas no hydrogen production was observed with pristine CoS_2_ nanoparticles. This result unveils that the metal dichalcogenide–CoS_2_ acts as an effective co-catalyst and nanocrystalline TiO_2_ serves as an active site by effectively separating the photogenerated electron–hole pair. This study lays down a new approach for developing transition metal dichalcogenide materials with significant bandgaps that can effectively harness solar energy for hydrogen production.

## 1. Introduction

Depletion of fossil fuel deserves utilization of hydrogen as a renewable energy source. It could be one of the promising energy sources alternative to fossil fuels in meeting the energy demand of the current world population. Currently, the major hydrogen production is from steam-methane reforming and thermal cracking of natural gas, and coal gasification, which cannot alleviate the emission of greenhouse gases [1]. Electrolysis is another method, which is being used to produce hydrogen. Electrochemical reduction of water [2,3,4] is an ecofriendly method and exhibits high-purity (99.999%) of hydrogen [5]; in addition, this is a key to many clean energy technologies. Although there have been several methods used to produce hydrogen, photocatalytic hydrogen generation have gained much attention these days. This technique integrates solar energy collection together with water splitting, therefore, it is a more cost-effective method compared with the water electrolysis process. Unfortunately, only a small percentage of hydrogen is produced from the photocatalytic method under solar extended irradiation. Therefore, the development of new materials for sustainable hydrogen production is necessary to overcome the detrimental environmental impacts. In the past decade, different photocatalyst materials, such as TiO_2_ [6,7,8], ZnO [9], CdS [10,11], WS_2_ [12,13], mixed oxides [14,15], perovskites [16,17], dye and metal doped oxide materials [18,19] have been used as phototcatalysts for environmental remediation and energy production, such as water splitting applications. In particular, catalysts contain noble metals, such as Pt have also been utilized in the state-of-the-art hydrogen evolution reaction. However, large scale hydrogen production is limited with these catalysts [20,21,22]. Among the materials studied, TiO_2_ has been considered as the golden standard due to its significant characteristics that include photochemical stability, low toxicity, relative affordability, and ease of preparation [23]. Although the bandgap energy of TiO_2_ necessitates the use of UV irradiation, composites of TiO_2_ with co-catalysts, such as transition metal chalcogenides (TMCs), enable the catalysts to absorb visible light abundant in solar irradiation [24]. In this regard, TMCs have gained much attention among the research community in the field of lithium ion batteries, solar cells and hydrogen evolution, due to their significant characteristic features that include indirect bandgaps, optoelectronic properties and stability [24]. In addition, nanodots (quantum dots)/nano structures of these metal chalcogenides show stronger edge effects, and the quantum confinement effect make them suitable to be utilized under solar simulated irradiation [24,25]. The transition metal chalcogenides can be synthesized by employing different techniques, such as one-pot wet chemical method [10], impregnation–sulfidation [11], simple microwave-assisted solvothermal process [26], ion exchange and precipitation methods [27], and hydrothermal method [28]. Several studies on bare transition metal chalcogenides (MoS_2_, NiS_2_, WS_2_, CdS and CoS) for the hydrogen evolution reaction (HER) and oxygen reduction reactions (ORR) are reported [29,30,31,32], however, most of these studies mainly focused on electrochemical water splitting. Co-catalysts, such as, reduced graphene oxides [33], metal oxides [34], dyes, for example, Porphyrin (Zn(II)-5,10,15,20-tetrakis(4-carboxyphenyl)-porphyrin (ZnTCPP)) [19], graphene [35], metals [36], and CdLa_2_S_4_ nanocrystals [37] were used to enhance the rate of HER. Inorganic crystals with desired properties can be used as excellent candidates for HER. It was reported that the first-row transition metal chalcogenides (MS_2_; where M = Fe, Co, Ni) exhibit excellent catalytic properties for HER due to their pyrite or marcasite structure, in which the metal atoms are octahedrally bonded to adjacent S atoms. Stability is an important criterion in the development of catalysts [30,38,39,40,41,42,43,44,45]. In this regard, computational modelling was also employed on these metal chalcogenide materials to evaluate their structural properties for photocatalytic water splitting and hydrogen production [46]. In particular, MoS_2_ has been utilized with other elements via adsorption or intercalation of a cation, such as Li for electrochemical water splitting [46]. In another modelling work, a comparative study was done between WS_2_ and MoS_2_ in electrocatalytic water splitting [12]; In an experimental study, Yuexiang Li et al. reported hydrogen production of 99 μmol h^−1^ with MoS_2_ loaded on the composites of reduced graphene oxide and CdS, and this was found to be over 20 times higher than the bare CdS. CoS_2_ doped with Mn [47] and Al [48] used for electrocatalytic HER [49].

Although all the reported work on TMC-embedded TiO_2_ mainly focused on the electrochemical water splitting and computer modelling, to the best of our knowledge, no experimental study on TMC-embedded TiO_2_ for heterogeneous hydrogen production over extended solar irradiation has been reported yet. This study focuses on the synthesis of CoS_2_ (metal chalcogenide)-embedded TiO_2_ nanocomposite, and the impact of doping CoS_2_ with TiO_2_ in hydrogen evolution under UV irradiation.

## 2. Materials and Methods

### 2.1. Materials

Without further purification, titanium isopropoxide, 98+% (Sigma-Aldrich Norway AS, Oslo, Norway) was employed as the precursor for TiO_2_ preparation, Cobalt (II) nitrate hexahydrate, 99% pure (Sigma-Aldrich Norway AS) was utilized as the cobalt precursor, and Na_2_S_2_O_3_ (Sigma-Aldrich Norway AS) as sulfur source. PHARMCO-AAPER Ethyl alcohol (200 Proof; Absolute, anhydrous, Sigma-Aldrich Norway AS) was used as solvent and deionized water (resistivity >18 Ω·cm, Velp/AREC, VELP Scientifica Srl, Usmate (MB), Italy) was used to prepare the solution mixtures.

### 2.2. Methods

#### 2.2.1. Synthesis

(1) Titanium dioxide

Nanocrystalline titanium dioxide material was prepared under hydrothermal condition using sol-gel technique. In a typical synthesis, 32.5 mL of ethanol was acidified with 0.3 mL concentrated HNO_3_ (Sigma-Aldrich, Oslo, Norway) in a Teflon liner and stirred at a constant speed (300 rpm, Velp/AREC, VELP Scientifica Srl,). 6.60 mL of titanium (iv) isopropoxide was added drop wise into it with continuous stirring, Finally, 3.0 mL of water was added to the above solution. Then, it was transferred into an autoclave (AUTOCLAVE-PTFE-0100, TECINSTRO, Maharashtra, India) and kept at 180 °C for 9 hours. The final material was heated at 500 °C for 3 h.

(2) CoS_2_ embedded TiO_2_ nanocomposite

118.24 mg of Co(NO_3_)_2_ and 192.72 mg of Na_2_S_2_O_3_ were added in to a 100 mL aqueous solution containing deionized water and ethanol in 2:1 ratio under constant stirring (300 rpm) for 30 min. Finally, required amount of TiO_2_ was dispersed into above solution and the resulting mixture was hydrothermally treated at 180 °C to prepare 10 wt.% of CoS_2_ embedded TiO_2_ material. Similar conditions were followed in the preparation of pristine CoS_2_ nanoparticles without adding titanium dioxide.

#### 2.2.2. Characterization

Synthesized materials were subjected to different characterization techniques, such as Powder X-ray Diffraction (P-XRD, Ultima IV Rigaku, USA) Method, Diffuse Reflectance Spectra (DRS Cary 100 Bio UV–Visible spectrophotometer, Santa Clara, CA, USA) and Scanning Electron Microscopy (SEM, Oxford instrument, NanoAnalysis, Concord, MA, USA). P-XRD patterns were recorded on a Rigaku Ultima IV instrument (Scottsdale, AZ, USA) with Cu Kα radiation (λ = 1.5408 Å) at ambient temperature, under the following operating conditions; accelerating voltage of 40 kV; emission current of 44 mA; scanned range (2θ) between 20° and 80° with a step size of 0.02°, and a scan speed of 1°/min. DRS were acquired using a Cary 100 Bio UV–Visible spectrophotometer, and the SEM images were captured on an Oxford instrument.

#### 2.2.3. Photocatalytic Hydrogen Evolution

The photocatalytic experiments were carried out for pristine TiO_2_, CoS_2_ and CoS_2_/TiO_2_. Catalysts were suspended in a solution containing 1.5 mL of H_2_O and 0.5 mL of methanol as a scavenging agent. The suspension was degassed for 30 minutes with high-purity argon prior to irradiation. The suspensions were continuously stirred throughout the course of the experiment. A 300 W Xenon lamp (Oriel light source, Xenon arc lamp, Newport 1000W, CA, USA) with an appropriate filter was used as the source of UV radiation. The amount of H_2_ produced was measured by gas chromatography (SRI 8610 C, SRI instruments, Torrance, CA, USA) equipped with a molecular sieve column and a TCD (Thermal Conductivity detector), and the amount of hydrogen produced was quantified by using a calibration curve prepared previously.

## 3. Results and Discussion

### 3.1. Characterization of Materials

**Figure 1:**
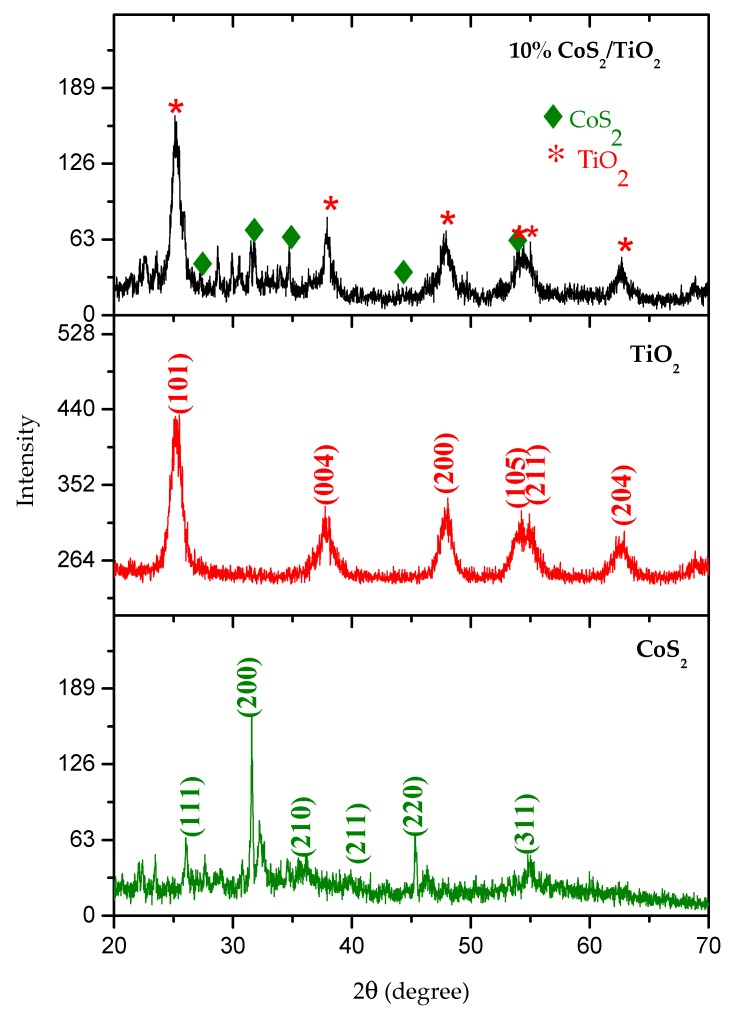
XRD patterns of the pristine CoS_2_, TiO_2_, and 10 wt.% CoS_2_/TiO_2_ nanocomposite.

The powder XRD patterns of the pristine CoS_2_, TiO_2_, and 10 wt.% CoS_2_/TiO_2_ nanocomposites are shown in Figure 1. The peaks observed at the 2 theta values of 26.04°, 31.58°, 37.08°, 40.34°, 45.34° and 55.1° are due to (111), (200), (210), (211), (220) and (311) diffraction planes of CoS_2_ (PDF Card No.: 9007682). The peaks at 2θ of 25.50°, 37.76°, 48.10°, 53.88°, 55.84° and 62.90° due to the (101), (004), (200), (105), (211) and (204) diffraction planes confirm the formation of TiO_2_ anatase phase (JCPDS 21-1272) [50]. Combination of CoS_2_ and TiO_2_ peaks observed with the 10 wt.% CoS_2_/TiO_2_ nanocomposite confirms good impregnation of CoS_2_ on TiO_2_.

**Figure 2:**
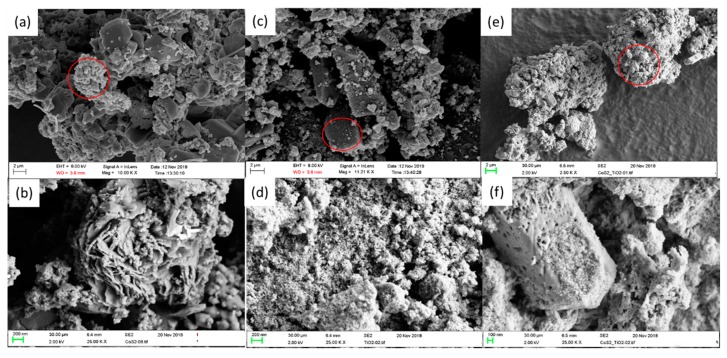
SEM images of the CoS_2_ (**a**,**b**), TiO_2_ (**c**,**d**), and 10 wt.% CoS_2_/TiO_2_ (**e**,**f**) nanocomposite.

Scanning electron microscopic images of blank CoS_2_ (a,b), TiO_2_ (c,d) and 10 wt.% CoS_2_/TiO_2_ (e,f) are illustrated in Figure 2. It can clearly be seen from Figure 2a,b that the bare CoS_2_ shows an aggregation, which is surrounded by flake like structures. An irregular 3D block-like structure covered with spongy like particles was attained for TiO_2_ nanocomposite and is shown in Figure 2c, and the zoom in image (Figure 2d) clearly illustrates the aggregated particles which have spongy-like structures. The mixed composite, CoS_2_/TiO_2_ also exhibits the aggregation, in which the zoom in image (Figure 2f) shows the hexagonal rod like structure decorated with spongy like materials [51].

**Figure 3:**
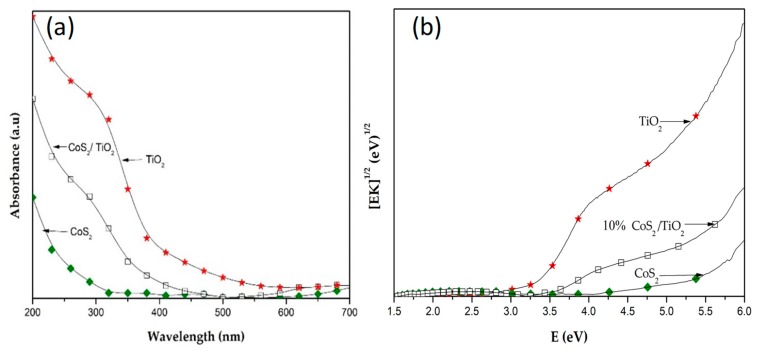
(**a**) Absorbance from diffuse reflectance spectra and (**b**) Tauc plots of pristine CoS_2_, TiO_2_ and 10 wt.% CoS_2_/TiO_2_ nanocomposite.

The bandgap energies for the pristine CoS_2_, TiO_2_ and 10 wt.% CoS_2_/TiO_2_ nanocomposite materials corresponding to the absorbance spectra of powder samples (Figure 3a), were estimated by using the Tauc plot (Figure 3b), which was transformed via the Kubelka–Munk function [52], ${\left[F\left(R_{\infty }\right)E\right]}^{n}$ vs. E, when n = 0.5, for a direct allowed transition $\left(K=F\left(R_{\infty }\right)\right)$. Estimates derived from the Tauc plots by extrapolating the steep portion of the plot in Figure 3b to the x-axis suggest that the bandgaps of the pristine CoS_2_ (2.5 eV), TiO_2_ (3.2 eV) and CoS_2_ embedded TiO_2_ nanocomposite (3.4 eV) materials lie in the range between 2.4 and 3.4 eV.

### 3.2. Hydrogen Evolution

The amount of hydrogen evolved in the presence of UV irradiation is tabulated and compared with what is reported in the literature in Table 1. Transition metal chalcogenides, including MoS_2_, NiS, SnS_2_, WS_2_, and CdS, have been extensively explored for photocatalytic water splitting [11,24,36,49], since they are usually inexpensive, stable, and easily prepared on a large scale for practical applications. To improve the efficiency of TMCs on hydrogen evolution elemental doping, heterojunctions, and nano structuring have been explored. In this regard, metal chalcogenides have been doped with other components, such as reduced graphene oxide, graphene, dyes, and TiO_2_ using different experimental conditions.

For example, Qun Wang et al. worked on MoS_2_ quantum dots-doped TiO_2_ for hydrogen evolution reaction in under different experimental condition, and the rate of hydrogen evolution was found to be 0.05 mmol cm^−2^ h^−1^ [24]. In another study, Youngjun Yuan et al. used ZnTCPP-MoS_2_ /TiO_2_ material for HER (Hydrogen Evolution Reaction), where 0.10 mmol h^−1^ of hydrogen evolved with 1.00 wt.% of MoS_2_ on TiO_2_ material [19]. In line with these studies, the results from our study on metal chalcogenide-TiO_2_ nanocomposites showed that 10 wt.% CoS_2_ embedded on TiO_2_ nanocomposites synthesized by hydrothermal method was the excellent candidate for the photocatalytic HER with better hydrogen evolution rate of 2.55 mmol g^−1^. Pristine CoS_2_ alone showed no activity towards hydrogen production even after 4 hours of irradiation, whereas the TiO_2_ materials exhibited 1.88 mmolg^−1^ of hydrogen under the same experimental conditions. The reason for this observation can be correlated with the bandgap energies of the materials. The CoS_2_/TiO_2_ materials with highest activity exhibit bandgap of 3.4 eV, whereas the pristine TiO_2_ exhibits bandgap of 3.2 eV. Under UV irradiation, the electron-hole pair formed on the nanocomposites was effectively separated due to the bandgap >3 eV. However, in the case of pristine CoS_2_ (Bandgap of 2.5 eV), it can be concluded that the faster recombination rate of photogenerated electrons and holes hinder the formation of hydrogen effectively, and, thus, there is no activity observed with this catalyst, but CoS_2_ nanoparticles act as a co-catalyst in the nanocomposite of CoS_2_/TiO_2_ materials to enhance the hydrogen production by exciting more electrons to the surface of titanium dioxide.

## 4. Conclusions

Pristine CoS_2_ and TiO_2_, and CoS_2_/TiO_2_ nanocomposites were successfully synthesized via a hydrothermal method using titanium(iv)isopropoxide, Co(NO_3_)_2_ and Na_2_S_2_O_3_ as precursors. The mixed COS_2_/TiO_2_ nanocomposite exhibits a high hydrogen production value of 2.55 mmol g^−1^, whereas the pristine CoS_2_ material was found to be inactive due to its very low bandgap energy. The TiO_2_ material shows an intermediate hydrogen production of 1.88 mmol g^−1^. In summary, the hydrogen production seems to depend on the band gap energy of the catalysts, and the CoS_2_ may assist to effectively separate the electron-hole pair forms in the mixed nanocomposite, and thus, results in a higher value of hydrogen production.

## Figures and Tables

**Table 1:** Amount of hydrogen evolved with different transition metal Chalcogenides: A comparison.

Material	Synthesis Method	Rate of Hydrogen Evolution	Sacrificial Agent	Reference	
2D SnS_2_/g-C_3_N_4_ (5 wt.% SnS_2_/g-C_3_N_4_)	Hydrothermal method	0.97 mmol h^−1^ g^−1^	10 vol% TEOA and 3 wt.% H_2_Pt_2_Cl_6_·6H_2_O	Enzhou Liu et al., 2018	[53]
Te/SnS_2_/Ag	Hydrothermal method	0.33 mmol h^−1^	-	Changzeng Yan et al., 2017	[36]
SnS_2_ Nanosheets	Solvothermal	1.06 mmol h^−1^ g^−1^	0.1 M Na_2_S 0.1M Na_2_S_2_O_3_	Jing yu et al., 2014	[54]
CdS/ WS_2_	Impregnation-sulfidation	0.42 mmol h^−1^	Latic acid solution	Zong et al., 2011	[11]
Dye-Sensitized NiS_x_/ graphene (in EY/G)	Insitu chemical deposition method	0.04 mmol h^−1^	-	Chao Kong et al., 2014	[55]
Dye-Sensitized NiS_x_/ graphene (in EY/NiS_x_/G)	Insitu chemical deposition method	0.34 mmol h^−1^	-	Chao Kong et al., 2014	[55]
MoS_2_/ RGO and CdS (pH11-MoS_2_/rGO 1.5/CdS)	Photoreduction method	0.10 mmol h^−1^	10 vol.% Latic acid solution	Yuexiang Li et al., 2014	[49]
MoS_2_/Graphene	Hydrothermal	1.80 mmol h^−1^	Na_2_S-Na_2_S_2_O_3_ solution	Chang et al., 2014	[35]
MoS_2_ quantum dots/TiO_2_ nanotube arrays	Electrodeposition	0.07 mmol cm^−2^ h^−1^ 0.05 mmol cm^−2^ h^−1^ 0.02 mmoL cm^−2^ h^−1^	-	Qun Wang et al., 2018	[24]
ZnTCPP-MoS_2_ /TiO_2_ (1.00 wt.% MoS_2_ on TiO_2_)	Hydrothermal	0.10 mmol h^−1^	0.2 M triethanolamine (TEOA) aqueous	Youngjun Yuan et al., 2015	[19]
10 wt.% CoS_2_/TiO_2_	Hydrothermal	2.55 mmol g^−1^	Methanol	This work	
